# TDP-43-Mediated Neuron Loss *In Vivo* Requires RNA-Binding Activity

**DOI:** 10.1371/journal.pone.0012247

**Published:** 2010-08-18

**Authors:** Aaron Voigt, David Herholz, Fabienne C. Fiesel, Kavita Kaur, Daniel Müller, Peter Karsten, Stephanie S. Weber, Philipp J. Kahle, Till Marquardt, Jörg B. Schulz

**Affiliations:** 1 Department of Neurology, University Medical Center, RWTH Aachen, Aachen, Germany; 2 Developmental Neurobiology Laboratory, European Neuroscience Institute-Göttingen, Göttingen, Germany; 3 Laboratory of Functional Neurogenetics, Department of Neurodegeneration, Hertie Institute for Clinical Brain Research and German Center for Neurodegenerative Diseases, Tübingen, Germany; 4 JARA Brain (Jülich-Aachen-Research-Alliance), Jülich, Germany; Brigham and Women's Hospital, Harvard Medical School, United States of America

## Abstract

Alteration and/or mutations of the ribonucleoprotein TDP-43 have been firmly linked to human neurodegenerative diseases, including amyotrophic lateral sclerosis (ALS) and frontotemporal lobar degeneration (FTLD). The relative impacts of TDP-43 alteration, mutation, or inherent protein function on neural integrity, however, remain less clear—a situation confounded by conflicting reports based on transient and/or random-insertion transgenic expression. We therefore performed a stringent comparative investigation of impacts of these TDP-43 modifications on neural integrity *in vivo*. To achieve this, we systematically screened ALS/FTLD-associated and synthetic TDP-43 isoforms via same-site gene insertion and neural expression in *Drosophila*; followed by transposon-based motor neuron-specific transgenesis in a chick vertebrate system. Using this bi-systemic approach we uncovered a requirement of inherent TDP-43 RNA-binding function—but not ALS/FTLD-linked mutation, mislocalization, or truncation—for TDP-43-mediated neurotoxicity *in vivo*.

## Introduction

TAR-DNA binding protein-43 (TDP-43) is a multifunctional heterogeneous ribonucleoprotein implicated in mRNA processing and stabilization [1]. TDP-43 comprises two RNA recognition motifs (RRMs), a nuclear localization signal and a nuclear export sequence mediating nuclear shuttling, as well as a C-terminal glycine-rich domain (GRD) implicated in TDP-43 protein interactions and functions [1]. Cytosolic accumulation of truncated TDP-43 is found in affected neurons of patients suffering from sporadic and familial ALS and FTLD [2], [3]. Moreover, missense mutations clustering in the TDP-43 GRD have been identified in many cases of ALS (and FTLD) [1], [4], [5]. The precise impacts of the missense mutations and/or aggregation of mutant, or wild type TDP-43 for neuropathology, however, remain unclear. Based on a number of recent *in vitro* and *in vivo* studies, both neurotoxic gain-of-function and loss-of-function mechanisms have been proposed linking TDP-43 proteinopathy to neuropathology [6], [7]. For instance, TDP-43 missense mutations have been suggested to mildly increase truncation and cytosolic localization/aggregation of the protein, and this has been correlated with increased cytotoxicity [8], [9]. The relationship between inherent protein function, ALS/FTLD-linked missense mutations (TDP-43^MS^), or truncation/mislocalization and TDP-43-mediated neuropathology *in vivo*, however, is less clear. This is further confounded by recent studies separately reporting that either overexpression of wild type TDP-43 (TDP-43^WT^), or ALS/FTLD-linked TDP-43^MS^, or inactivating endogenous TDP-43 [10], [11], [12], [13], [14] all lead to neuron loss *in vivo* in different invertebrate and vertebrate models [10], [11], [12], [13], [15], [16]. In transgenic mice, for instance, forced expression of TDP-43^WT^ and human ALS/FTLD-linked TDP-43 variant TDP-43^A315T^ phenocopied pathological hallmarks of TDP-43-linked ALS [12]. Recently, mutations in another ribonucleoprotein FUS have been identified in ALS cases [17] suggesting possible commonalities in mechanisms of these two RNA-binding proteins that link TDP-43 and FUS to neurodegeneration [1], [7]. This further raises the question to what extent the inherent physiological activities exerted by TDP-43 contribute to the toxic properties observed upon TDP-43 overexpression in the different model systems. At this stage, a priority in the field should therefore be the clarification of the relative impacts of inherent TDP-43 protein function and ALS/FTLD-linked mutation/alteration on neurotoxicity mediated by TDP-43 expression *in vivo*.

## Results

### Stringent comparative assessment of TDP-43 mutation and alteration via same-site controlled expression

**Figure 1:**
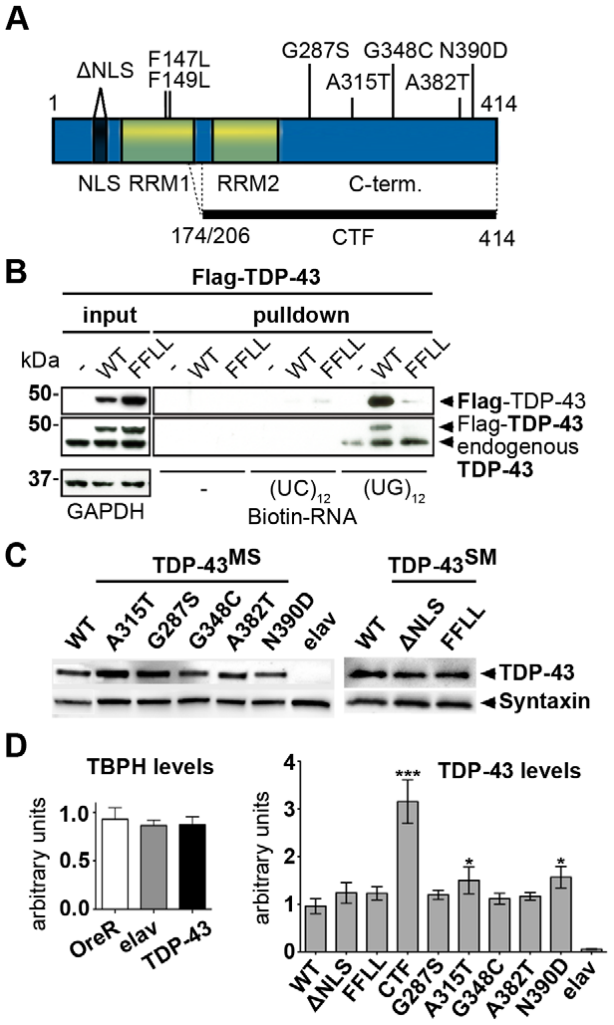
Analysis of different TDP-43 variants used for ectopic expression. (A) Schematic overview of TDP-43 variants tested *in vivo*. Positions and nature of amino acid substitutions and size of two C-terminal fragments (CTFs) analyzed are indicated. Synthetic mutations (SM) were introduced to interfere with TDP-43 localization or function. Mutations in the nuclear localization signal (ΔNLS) were introduced to target TDP-43 to the cytoplasm and mutations F147L/F149L (FFLL) in the first RNA recognition motive (RRM1) were introduced to impair TDP-43 inherent RNA-binding capacity. (B) Loss of RNA-binding by TDP-43^FFLL^. Biotinylated oligonucleotides were incubated with lysates from either untransfected HEK293 cells or HEK293 cells transfected with FLAG-tagged TDP-43^WT^ or TDP-43^FFLL^ (left, input) followed by UV crosslinking and streptavidin-pulldown of Biotin-RNA (right, pulldown). Precipitates were separated, blotted and membranes probed with FLAG- (upper panel) and TDP-43-specific (lower panel) antibodies to assay co-precipitation of TDP-43. Note: Co-precipitation of endogenous TDP-43 with (UG)_12_-repeats in all lysates. Ectopic FLAG-TDP-43^WT^, but not FLAG-TDP-43^FFLL^ co-precipitated with cognate UG repeats. GAPDH served as loading control for protein input. (C) Equalized pan-neural expression of *φ-C31*-inserted TDP-43 variants in adult *Drosophilae*. TDP-43 expression was visualized using an anti-human TDP-43 antibody. Syntaxin served as loading control. (D) Assessment of relative TBPH and TDP-43 expression levels. Left graph: Relative abundance of endogenous TBPH transcripts in relation to *actin5C* of wild type strain OregonR (OreR), the Gal4-driver line (*elav^C155^::Gal4*) and flies with pan-neural TDP-43 expression (*elav^C155^::Gal4/Y;UAS::TDP-43^WT^/+*). TBPH levels were not significantly different in analyzed genotypes. Right graph: mRNA abundance of TDP-43 in flies with pan-neural expression of the different TDP-43 variants normalized to *actin5C* and *TBPH* mRNA levels. Significant differences are indicated. *p<0.05; ***p<0.001. Head lysates of flies with pan-neural expression of TDP-43 were used for Western blot and qPCR analysis. *elav::Gal4* flies without a TDP-43 transgene (elav) were used as negative control.

We reasoned that a first step to clarify the role of TDP-43 in ALS and FTLD would require a systematic and direct comparative investigation of respective disease-linked variants of TDP-43 [4] on neural integrity *in vivo*—to alleviate seemingly conflicting conclusions drawn from separate experiments addressing different TDP-43 variants in diverse systems at varying expression levels. To achieve this, we generated expression constructs for a series of TDP-43 variants, including TDP-43^WT^, synthetic mutants (TDP-43^SM^) and ALS/FTLD-linked TDP-43^MS^ (Fig. 1A). To analyze possible effects of TDP-43 mislocalization, we introduced a synthetic protein modeled after the major TDP-43 C-terminal fragment (TDP-43^CTF^) found in cytosolic aggregates of ALS/FTLD patients [3], as well as intra-cellularly mislocalized full-length TDP-43 lacking a functional nuclear localization signal (TDP-43^ΔNLS^). TDP-43 is known to effectively bind RNA [18], [19] and this has been shown to result in stabilization of bound mRNAs [20]. To investigate, whether this intrinsic function of TDP-43 might be crucial for TDP-43-mediated neurotoxic action, we introduced two missense mutations (F_147_L/F_149_L) into the first RNA recognition motif (RRM1) to abolish inherent TDP-43 RNA-binding function [21]. In contrast to TDP-43^WT^, TDP-43^FFLL^ displayed a complete loss to bind to UG_12_ RNA oligomers in pull-down assays (Fig. 1B).

To comparatively analyze the effect of neural expression of the respective human TDP-43 variants *in vivo*, we generated transgenes utilizing *φ-C31* site-specific recombination in *Drosophila* [22], [23]. Expression of the transgenic constructs is controlled by the UAS/Gal4 system, allowing targeted expression of TDP-43 in a spatiotemporal manner [24]. To avoid possible interference of protein-tag fusions with TDP-43 activity, we utilized ‘untagged’ proteins and verified their expression and relative protein levels (Fig. 1C). Although we cannot entirely rule out the possibility that the introduced mutations to some extent alter protein turnover/stability, we were not able to detect obvious differences in the expression levels of the different TDP-43 variants by Western blot analysis. TDP-43^CTF^ lacks large portions of the epitope/s detected by the available anti-TDP-43 antibody which results in no/weak recognition of TDP-43^CTF^ compared to full length protein in Western blot analysis [25]. For this reason, we assessed relative TDP-43^CTF^ expression by quantitative RT-PCR (qRT-PCR) analysis (Fig. 1D). First we analyzed transcript abundance of both human TDP-43 and endogenous TBPH (fly homolog of TDP-43) by qRT-PCR (Fig. 1D). The relative abundance of TBPH transcripts was not significantly altered between non-transgenic (OreR), transgenic (*elav^C155^::Gal4*) controls and TDP-43^WT^ expressing flies, indicating that TDP-43 expression had no detectable impact on endogenous TBPH transcript levels in these flies. Next we performed qRT-PCR of all TDP-43 variants normalized to endogenous *actin5C* and *TBPH*. This analysis revealed significantly higher transcript levels for TDP-43^CTF^. Although TDP-43^A315T^ and TDP-43^N390D^ expression levels were found to be slightly, but significant elevated compared to that of TDP-43^WT^, the overall expression levels of TDP-43^MS^ variants were similar (Fig. 1D).

### Subcellular localization of TDP-43 variants *in vitro* and *in vivo*

**Figure 2:**
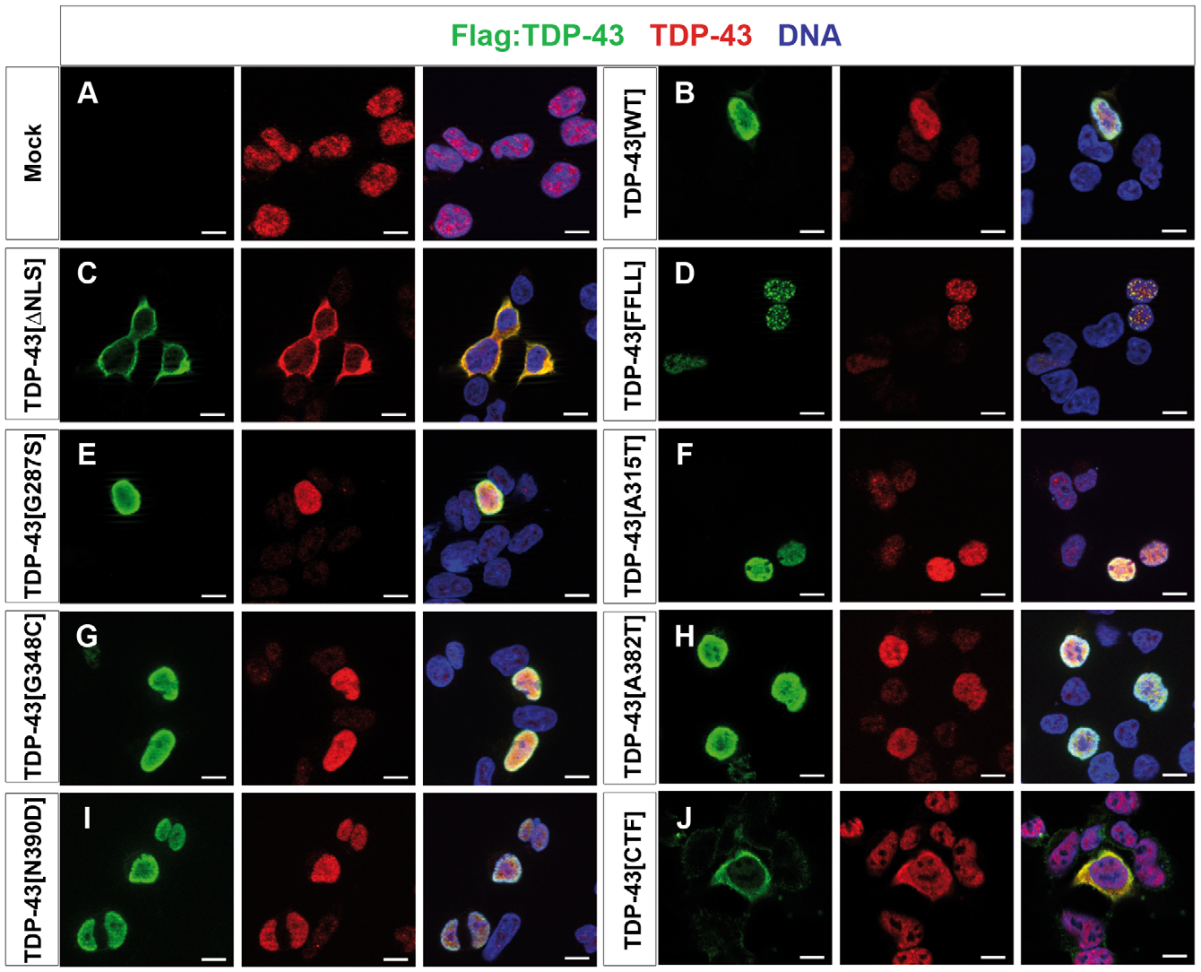
Localization of TDP-43 variants in human cells. HEK293E cells transfected with N-terminal Flag-tagged TDP-43 variants detected with Flag- (left, green) and TDP-43 (middle, red) specific antibodies. Overlay (right) with Hoechst stained DNA (blue). (A) Exogenous TDP-43 had to be visualized via fused Flag-tag, as HEK cells show robust endogenous expression of TDP-43. (B) Ectopic TDP-43^WT^ mainly localized to the nucleus. (C) In contrast, TDP-43^ΔNLS^ exclusively localized to cytosol without showing nuclear Flag-signal. Similar to TDP-43^FFLL^ (D), also TDP-43^MS^ (E–I) displayed a nuclear localization. However, TDP-43^FFLL^ localized in a characteristic punctuate pattern throughout the nucleus (D). (J) TDP-43^CTF^ displayed a predominantly cytoplasmic localization similar to TDP-43^ΔNLS^ (compare C and J). Scale bar indicates 10 µm.

**Figure 3:**
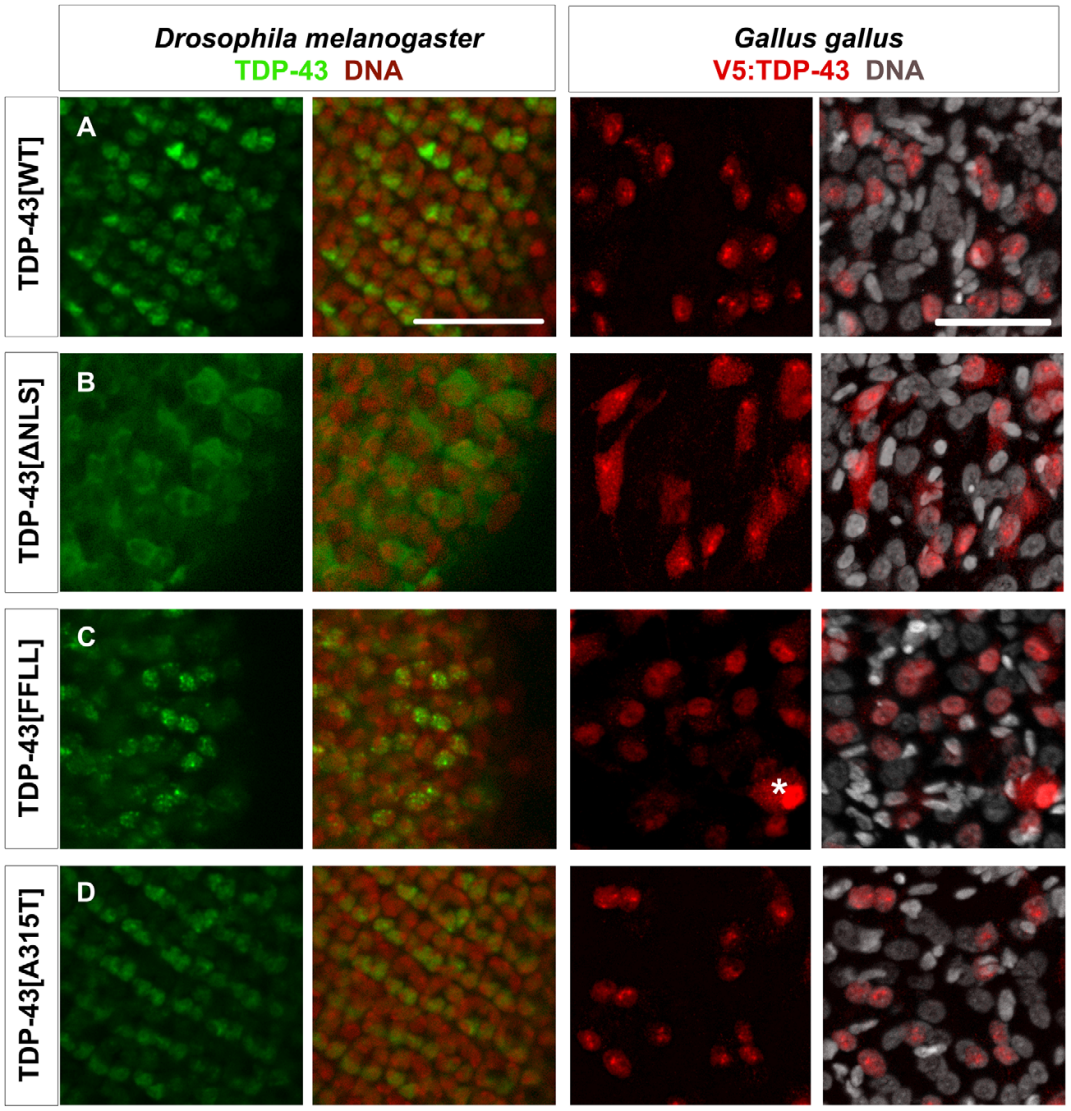
Localization of TDP-43 in *Drosophila melanogaster* and *Gallus gallus*. Confocal sections of eye imaginal discs from *Drosophila* larvae (left panel) and motor neurons from *Gallus* (right panel) expressing indicated TDP-43 variants. To be able to discriminate between the two *in vivo* systems, ectopic TDP-43 in *Drosophila* is shown in green, whereas TDP-43 in *Gallus* is shown in red. Subcellular localization of the different TDP-43 variants was found to be identical between fly and chick. TDP-43^WT^ (A) localized mainly to the nucleus, while TDP-43^ΔNLS^ (B) was found predominantly in the cytoplasm. TDP-43^FFLL^ (C) and TDP-43^A315T^ (D) displayed a nuclear distribution. Only cells with very high expression levels of usually nuclear TDP-43 displayed a detectable cytoplasmic staining (example in case of TDP-43^FFLL^ marked by asterisk). DNA was stained with Sytox® Orange (fly, red) or DAPI (chick, white). Scale bar indicates 50 µm. Neuronal expression was mediated by *elav::Gal4* (flies) or *Hb9::Cre* (chick).

We next tested the subcellular localization of the different TDP-43 variants in human embryonic kidney (HEK) cells (Fig. 2), non-neuronal *Drosophila* cells (Fig. S1), as well as in neuronal *Drosophila* cells and chick motor neurons (Fig. 3). The different TDP-43 variants tested displayed consistent subcellular localization patterns in these different cell types (Fig. 2; Fig. 3; Fig. S1). In all systems tested, ectopic TDP-43^WT^ mainly localized to the nucleus (Fig. 2B; Fig. 3A; Fig. S1A), while TDP-43^ΔNLS^ displayed predominant cytosolic localization (Fig. 2C, 3B, S1B), thus demonstrating efficient disruption of the nuclear localization signal in this TDP-43 variant. The second synthetic TDP-43 variant, RNA-binding-deficient TDP-43^FFLL^, predominantly localized to the nucleus and displayed a speckle-like pattern consistent with previous reports [26] (Fig. 2D; Fig. 3C; Fig. S1C). Similar to TDP-43^WT^, TDP-43^A315T^ (Fig. 2F; Fig. 3D; Fig. S1E), and all other TDP-43^MS^ tested mainly localized to the nucleus (Fig. 2E–I; Fig. S1D–H). TDP-43^CTF^ which lacks the NLS, displayed markedly cytoplasmic localization in HEK cells and chick motor neurons, with occasional localization to extranuclear foci (Fig. 2J; Fig. S3A). Similarly, GFP-tagged TDP-43^CTF^ displayed cytoplasmic localization in *Drosophila* neurons *in vivo* (Fig. S2C).

### Pan-neural expression of TDP-43 reduces longevity in *Drosophila*

**Figure 4:**
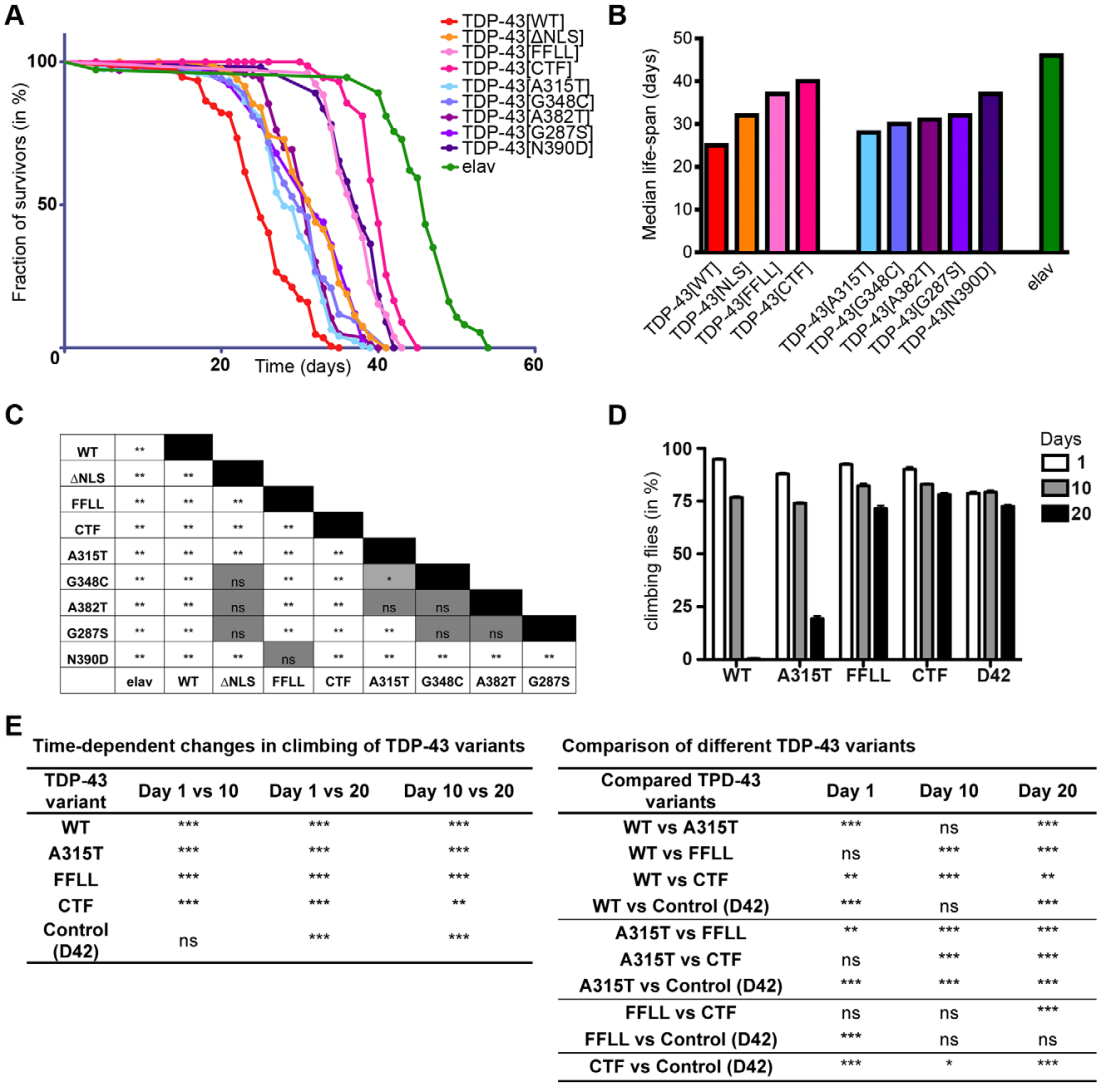
Requirement for RNA-binding activity for TDP-43-mediated neural defects in *Drosophila*. (A) TDP-43 expression reduces longevity in *Drosophila*. Flies with pan-neural (*elav*::*Gal4*) expression of indicated TDP-43 variants were assayed for longevity. (B) Median survival of respective survival curves. (C) Cross-comparison of survival curves with regard to statistical significance after Bonferroni correction. (D) Age-dependent locomotion defects after TDP-43 expression in motor neurons. Flies expressing indicated TDP-43 variants specifically in motor neurons (*D42::Gal4*) were assayed for negative geotaxis at 1, 10 and 20 days post eclosion. (E) Detailed summary of statistical analysis (2-Way ANOVA followed by Bonferroni post-hoc tests) of age-dependent locomotion effects. Genotypes of flies analyzed: *elav^C155^::Gal4/Y;UAS::TDP-43/+* in longevity analysis and *w/Y;UAS::TDP-43/+;D42::Gal4/+* in case of locomotion assay. *elav^C155^::Gal4/Y* (longevity) and *w/Y;;D42::Gal4* (climbing) served as controls. *p<0.05; **p<0.01; ***p<0.001; ns not significant.

We next analyzed the effect of ubiquitous and pan-neural expression of all TDP-43 variants on viability (Table 1). With the exception of TDP-43^CTF^ and TDP-43^FFLL^, ubiquitous expression of all TDP-43 variants tested resulted in premature lethality (Table 1). Similarly, pan-neural expression of most TDP-43 variants resulted in shortened life-span compared to control animals (Fig. 4A). However, comparison among TDP-43 expressing flies revealed profound variant-specific differences in relative impacts on longevity (Fig. 4A). TDP-43^WT^ expressing flies consistently displayed the most drastic reduction in life-span, while TDP-43^CTF^ and TDP-43^FFLL^ displayed comparatively mild reduction on longevity. Moreover, relative impacts on survival differed among the TDP-43^MS^ expressing flies: TDP-43^N390D^ displaying the lowest, TDP-43^A315T^ the highest reduction in median survival (Fig. 4B). A detailed statistical cross-correlation analysis revealed highly significant differences between the impacts of both, TDP-43^WT^ and TDP-43^CTF^ to all TDP-43 variants on longevity (Fig. 4C). In contrast, longevity curves of flies expressing other TDP-43 variants displayed less significant, or no significant differences to at least one other curve in the cross-correlation after Bonferroni correction (Fig. 4C).

**Table 1:** Expression of TDP-43 variants.

TDP-43 transgenes	Gal4-driver lines used for assay	
Site-directed insertions	*elav^C155^::Gal4* (pan-neural)	*actin5C::Gal4* (ubiquitous, strong)
TDP-43^WT^	vital	lethal
TDP-43^ΔNLS^	vital	lethal
TDP-43^FFLL^	vital	vital
TDP-43^G287S^	vital	lethal
TDP-43^A315T^	vital	lethal
TDP-43^G348C^	vital	lethal
TDP-43^A382T^	vital	lethal
TDP-43^N390D^	vital	lethal
TDP-43^CTF^	vital	vital

Expression of TDP-43 variants using different Gal4-driver lines. Impact of TDP-43 expression on vitality was assayed.

### Motor neuron-specific expression of TDP-43 impairs locomotion

We observed that TDP-43-induced lethality was preceded by progressive impairment of locomotor activity and wing posture (Video S1). To further investigate this, we utilized *D42::Gal4*-mediated motor neuron-specific expression, followed by quantitative assessment of motor performance through climbing assays (Fig. 4D,E). At day one of the analysis, roughly 80–90% of tested flies managed the climbing task. The slight reduction in climbing ability of control flies (D42) compared to TDP-43 expressing flies at day one of the analysis was unexpected. However, climbing ability of control flies remained stable at day 10 and showed only a slight reduction after 20 days. In contrast, expression of TDP-43^WT^ and TDP-43^A315T^ led to significant progressive loss of motor performance, with TDP-43^WT^ showing the highest reduction (Fig. 4D,E), in line with the observed relative impacts of these TDP-43 variants on longevity. Although still alive, 20 days old TDP-43^WT^ expressing flies appeared paralytic, hardly showed coordinated movement and failed to climb. Similar to control flies, TDP-43^CTF^ and TDP-43^FFLL^ expressing flies showed a slight reduction in locomotion over time (Fig. 4D), which most likely reflects normal aging rather than detrimental effects of TDP-43 as seen in TDP-43^WT^ and TDP-43^A315T^ expressing flies. Together, these results thus reveal a requirement of TDP-43 RNA-binding activity for TDP-43 mediated toxicity *in vivo.* Expression of both variants, TDP-43^FFLL^ and TDP-43^CTF^, lacking the first RRM resulted in much weaker detrimental effects on longevity and locomotion than TDP-43 variants with intact RRM1. At present, however, we cannot strictly exclude that TDP-43^FFLL^ may retain residual RNA/DNA binding activity exerted by the remaining first RRM, which may underlay the slightly higher toxicity of TDP-43^FFLL^ compared to TDP-43^CTF^.

### Dose-dependency of TDP-43-mediated neurotoxicity

In addition to our analysis of the same-site TDP-43 transgenes, we further generated transgenes expressing GFP-tagged TDP-43 variants (TDP-43^WT^:GFP, TDP-43^ΔNLS^:GFP and TDP-43^CTF^:GFP) through random insertion transgenesis. In flies expressing these variants, the intracellular localization of TDP-43 mirrored that observed for the corresponding untagged TDP-43 variants (Fig. S2). Since these lines displayed great differences in expression levels, they allowed us to investigate possible dose-dependency of TDP-43-mediated effects upon pan-neural and ubiquitous expression (Table 2). Comparison of two TDP-43^WT^:GFP lines with different expression levels showed a correlation between expression strength and longevity, with higher expression levels inducing an earlier lethality upon pan-neural expression (Fig. S4A). This indicated that TDP-43-mediated effects were dose-dependent, which was further suggested by the observation that two copies of *UAS::TDP-43^WT^*-transgenes caused lethality after pan-neural expression, whereas one copy did not (not shown). Moreover, we identified one TDP-43^WT^:GFP line to cause lethality after pan-neural expression. However, this line (TDP-43^WT^:GFP#14) did not display a lethal phenotype after muscle or glia-specific expression (Table 2), suggesting that effects after pan-neural expression of TDP-43 were cell-intrinsic.

**Table 2:** Expression of TDP-43:GFP variants.

TDP-43 transgenes	Gal4-driver lines used for assay			
Random insertions:	*elav^C155^::Gal4* (pan-neural)		*DaG32::Gal4* (ubiquitous, weak)	
TDP-43^WT^:GFP (11 lines)	10 vital, 1 lethal (line #14, used for further analysis)		1 vital,10 lethal	
TDP-43^ΔNLS^:GFP (16 lines)	15 vital, 1 lethal		1 vital, 15 lethal	
TDP-43^CTF^:GFP (11 lines)	11 vital, 0 lethal		11 vital, 0 lethal	
	***ey::Gal4*** ** (eye)**	***repo::Gal4*** ** (glia)**	***MHC::Gal4*** ** (muscle)**	***actin5C::Gal4*** ** (ubiquitous)**
TDP-43^WT^:GFP #14	vital	vital	vital	lethal

Expression of TDP-43:GFP variants using different Gal4-driver lines. Impact of TDP-43 expression on vitality was assayed.

Next, we compared the impact of ubiquitous (*DaG32*::*Gal4*) expression of TDP-43^WT^:GFP, TDP-43^ΔNLS^:GFP and TDP-43^CTF^:GFP on adult viability (Table 2). Almost no adult offspring was obtained after ubiquitous expression of TDP-43^WT^:GFP (10/11 independent lines) and TDP-43^ΔNLS^:GFP (15/16 independent lines). Ubiquitous expression of TDP-43^CTF^:GFP however, was never observed to cause lethality (0/11 independent lines). Even lines with markedly higher TDP-43^CTF^:GFP expression levels than TDP-43^WT^:GFP did not result in a comparable decline in adult viability, suggesting that TDP-43^CTF^:GFP, similar to untagged TDP-43^CTF^, does not exert robust toxicity (Table 2, Fig. S4A). Thus, the relatively mild reduction in longevity and climbing in previous experiments was most likely not related to TDP-43^CTF^ protein levels but rather appeared to reflect low toxicity mediated by TDP-43^CTF^ *per se*.

To exclude that the observed reduction in longevity was due to a developmental phenotype, we next used an adult-onset expression system, utilizing a temperature sensitive inhibitor of Gal4, Gal80^ts^. Under restrictive conditions, transgene expression was effectively silenced, but switched on within hours after shifting flies to permissive conditions, and induced TDP-43 expression was maintained for days (Fig. S4B). Intriguingly, pan-neural expression of untagged TDP-43^WT^ in this assay also caused a strong reduction in longevity, as compared to other TDP-43 variants (Fig. S4C). These data indicate that TDP-43-induced reduction in longevity was not dependent on developmental alterations of the nervous system.

### TDP-43-mediated motor neuron loss in chick mirrors relative impacts of TDP-43 mutation/mislocalization in *Drosophila*

**Figure 5:**
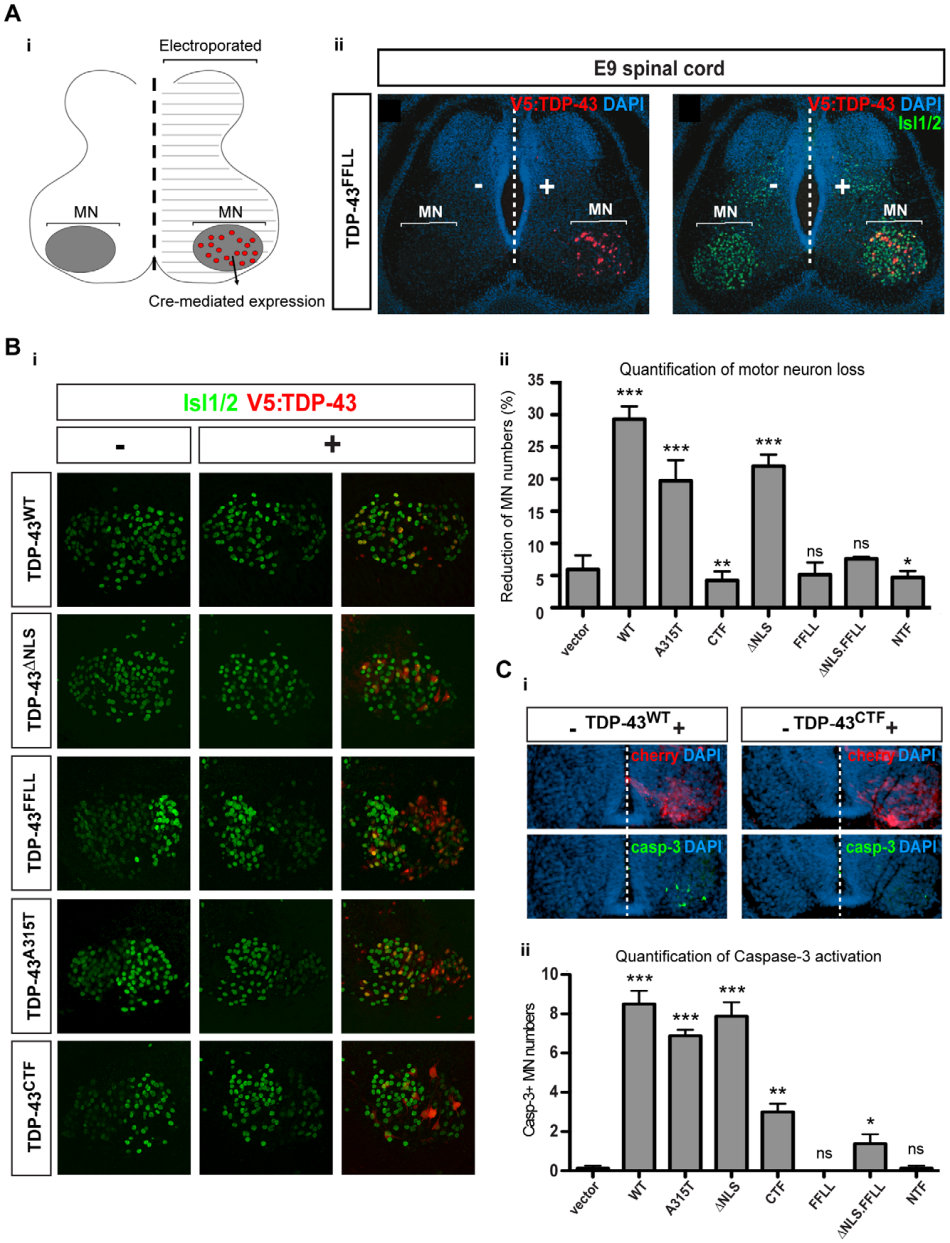
RNA-binding activity is required for TDP-43-mediated motor neuron loss in chick. (A) Stable unilateral expression of human TDP-43 variants in chick (*Gallus gallus*) spinal cord. [i] Schematic of expression system mediating motor neuron-restricted expression upon unilateral transfection. [ii] Unilateral expression of human TDP-43^FFLL^ (red) in embryonic day 9 (E9) chick spinal cord (nuclei labeled with DAPI: blue): transversal section at lumbar levels. “−” and “+” respectively indicate control and transfected hemicords. Isl1/2 labels motor neuron nuclei (green). (B) [i] Examples of thoracic motor columns (Isl1/2^+^ motor neurons: green) upon TDP-43 variant expression (red). [ii] Quantification of motor neuron loss upon TDP-43 variant expression over all obtained sections (in “-” versus “+” hemicord). Differences relative to control (t-student's test) are indicated. (C) [i] Activated Caspase-3 (green) detected in E5 motor neurons upon TDP-43^WT^ expression (indicated by *IRES-cherry* bi-cistronic reporter: red). Compared to E5 motor neurons expressing TDP-43^WT^, little activation of Caspase-3 was detected upon TDP-43^CTF^ expression. [ii] Quantification of Caspase-3 activation in motor neurons of transfected hemicords versus vector control. Significant differences are indicated (t-student's test - relative to control). *p<0.05; **p<0.01; ***p<0.001; ns not significant.

We next investigated whether the relative activities of TDP-43 variants found in *Drosophila* would also manifest themselves in a vertebrate system. We focused on the chick as a model, taking advantage of the temporally compressed neuromuscular maturation typical for precocial species. This model system thus facilitates ready experimental access to mature vertebrate motor neurons through *in ovo* transfection. To investigate impacts of TDP-43 expression in chick motor neurons we employed a novel expression system achieving stable neuron subtype-specific transgene expression in chick *in vivo* (DM, DH, TM, unpublished). This was based on *tol2*-transposon-mediated stable genomic insertion [27], combined with restricted transgene-activation via postmitotic motor neuron-specific Cre-recombination mediated by an *Hb9::Cre* driver construct. This system facilitated stable N-terminally V5 peptide-tagged TDP-43 protein expression in late-stage chick motor neurons (Fig. 5A). Intracellular localization of the different TDP-43 variants in chick motor neurons accurately mirrored that observed for the corresponding protein variants in HEK cells (Fig. 2) and *Drosophila* non-neuronal (Fig. S1) and neuronal cells (Fig. 3). Unilateral expression of TDP-43^WT^ in postmitotic motor neurons for 6 days triggered loss of roughly 30% Isl1/2^+^ spinal motor neurons relative to the untransfected control hemicord (Fig. 5B). Significant, but comparatively milder motor neuron loss was observed upon expression of TDP-43^ΔNLS^ and TDP-43^A315T^. Loss of motor neurons by TDP-43^WT^, TDP-43^A315T^ and TDP-43^ΔNLS^ was further preceded by transient increase in Caspase-3^+^ motor neuron numbers, mirroring the relative impact of TDP-43 variants on overall reduction of motor neurons numbers (Fig. 5C). This suggested that motor neuron loss occurred at least in part via caspase-dependent apoptotic elimination.

### TDP-43-mediated motor neuron loss in chick requires RNA-binding function

Expression of TDP-43^CTF^ (lacking the N-terminal portion including RRM1) or RNA-binding deficient TDP-43^FFLL^, in contrast, did not trigger enhanced motor neuron loss compared to control vector transfection (Fig. 5B). These data therefore suggested a requirement for RNA-binding activity for effective TDP-43-mediated neurotoxicity in chick, analogous to the relative inertness of TDP-43^FFLL^ and TDP-43^CTF^ regarding longevity and motor function in *Drosophila* (Fig. 4). In addition, these observations revealed that protein truncation/mislocalization may have comparatively little impact on TDP-43-mediated neurotoxicity, since TDP-43^CTF^ and TDP-43^FFLL^ displayed predominantly cytoplasmic or nuclear localization, respectively. To further test this, we designed cytosolically localized *plus* RNA-binding deficient TDP-43^FFLL,ΔNLS^ to separate relative impacts of truncation/mislocalization from inherent TDP-43 protein function (Fig. S3B). Similar to TDP-43^FFLL^ and TDP-43^CTF^, TDP-43^FFLL,ΔNLS^ did not measurably trigger motor neuron loss compared to control transfections (Fig. 5B). However, for both TDP-43^CTF^ and TDP-43^FFLL,ΔNLS^ we observed a transient mild increase in Casp-3^+^ motor neurons at 2 days after transfection (Fig. 5C); which, however, was apparently insufficient to result in measurable reduction of motor neuron numbers at later stages (Fig. 5B). While overexpression of mislocalized RNA-binding deficient TDP-43 may therefore to a degree impact neural integrity, this effect appeared negligible when directly compared to that exerted by similar levels of TDP-43 variants retaining inherent RNA-binding function.

Most ALS/FTLD-linked missense mutations that have been mapped so far localize to the C-terminal GRD of TDP-43 (see Fig. 1A) (http://www.molgen.ua.ac.be/FTDmutations). We therefore tested the impact on exogenous TDP-43-triggered motor neuron loss upon removing the C-terminal portion of TDP-43. To achieve this we introduced into chick motor neurons a synthetic TDP-43 variant—TDP-43^NTF^: TDP-43 “N-terminal fragment”—and tested for both, induction of early apoptosis and eventual effects on motor neuron numbers (Fig. 5B,C). Similar to TDP-43^WT^ and the TDP-43^MS^, TDP-43^NTF^ predominantly localized to the nucleus (Fig. S3C). However, in contrast to TDP-43^WT^ and similar to TDP-43^FFLL^ and TDP-43^CTF^, TDP-43^NTF^ expression had no significant impacts on maintenance of normal motor neuron numbers (Fig. 5B), nor on motor neuron apoptosis levels (Fig. 5C). Thus, motor neuron loss mediated by exogenous TDP-43 expression requires both, N-terminally localized inherent RNA-binding function, as well as the integrity of the C-terminal section—suggesting the intriguing possibility that C-terminal TDP-43 missense mutations may act through a pathway determined by inherent TDP-43 RNA-binding function.

## Discussion

This study provides the first direct comparison of the respective activities of ALS/FTLD-associated variants relative to wild type TDP-43 with regard to their impacts on neural integrity. By clarifying the relative impacts of TDP-43 alteration and missense mutation these results provide important ramification for understanding the link between TDP-43 function and neuropathology. First, equalized TDP-43 protein expression levels in *Drosophila* revealed a requirement for inherent TDP-43 RNA-binding activity for eliciting neural defects. Second, elicitation of neural defects by TDP-43 expression appeared to be independent from ALS/FTLD-linked mutations, truncation, or mislocalization, all of which appeared to at least slightly reduce impacts caused by same-level expression of TDP-43^WT^. Third, transposon-mediated transgenesis in chick revealed TDP-43-mediated loss of spinal motor neurons. In this model we observed a dependency of TDP-43-mediated neurotoxicity on RNA-binding activity, but not mislocalization, truncation, or missense mutations, therefore accurately mirroring the results obtained by same-level TDP-43 expression in *Drosophila*. Together, these data are inconsistent with models based on a simple gain-of-function effect by TDP-43 missense mutation and/or truncation that would render expression of the protein neurotoxic.

In our *Drosophila* model, relative impacts of TDP-43 mutation and modification on TDP-43^WT^-mediated neural deficiency ranged from mild (A315T) to drastic (N390D; FFLL). This further highlights the importance of stringently controlling relative protein levels when investigating the activity of different TDP-43 isoforms. Accordingly, less overt variant-specific differences in toxicity could consequently be masked, or misrepresented, by varying TDP-43 concentrations—such as achieved by random-insertion transgenesis. For instance, low TDP-43^WT^ expression levels had only mild impacts on longevity, while high levels of TDP-43^WT^ resulted in a marked reduction of life-span (see Fig. S4A). Moreover, while our parallel random-insertion transgenes detected clear differences between impacts on overall viability respectively induced by TDP-43^WT^ and TDP-43^CTF^ expression, more subtle differences between TDP-43^WT^ and TDP-43^ΔNLS^—obvious in our *φ-C31* site-specific transgenes—were not resolved. Previous difficulties in normalizing relative toxicity to relative protein levels could therefore account for conflicting results separately showing toxic effects by either wild type, or ALS/FTLD-linked mutant TDP-43 *in vivo*.

What could be the link between TDP-43 RNA-binding activity and manifestation of neuropathology in ALS/FTLD? While this study focused on *in vivo* consequences of introducing exogenous proteins, our results are consistent with the notion that detrimental effects of exogenous TDP-43 expression could result from interference with its endogenous function/s involving RNA-binding. This could in turn reflect inherent dosage-sensitivity of vital processes controlled by endogenous TDP-43/TBPH towards alterations in TDP-43/TBPH protein levels—and could account for the similar neural impacts separately obtained by TDP-43 overexpression and knock down *in vivo* [10], [11], [12], [13], [15], [16]. In analogy, the observed reduction, rather than increase in TDP-43 neurotoxic activity by ALS/FTLD-linked mutations (this study) – possibly reflecting reduction of inherent protein function – could consequently point to a loss-of-function mechanism by which depletion of TDP-43 function (e.g. via missense mutation, and/or truncation/aggregation, followed by cytosolic sequestration) would eventually lead to neuron loss [6], [7], [16]. Our observation that in chick, motor neuron loss mediated by exogenous TDP-43 expression requires the integrity of the TDP-43 C-terminal section—in addition to its N-terminally localized RNA-binding function—could further point to the possibility that ALS/FTLD-linked C-terminal TDP-43 missense mutations may act through a pathway determined by inherent TDP-43 RNA-binding function. While future studies will have to further address these possibilities, our results stress the urgency to identify the normal physiological roles of TDP-43 *in vivo*, and their potential contribution to sustaining neural integrity. Our results further stress the potential pitfalls of entirely relaying on gain-of-function approaches to model TDP-43 proteinopathy *in vivo*. The challenge now lies in the development of appropriate *in vivo*-models that more accurately mirror TDP-43 proteinopathy mechanisms occurring in human ALS and FTLD. Together, our data resolve the relative impacts of TDP-43 mutation/alteration and implicate inherent TDP-43 RNA-binding function in mediating neuronal loss *in vivo*. This study should therefore provide an essential entry point for eventually determining the definitive mechanisms and pathways linking TDP-43 to human neuropathology.

## Methods

### Ethics Statement

According to the German “Tierschutzgesetz” (BGBl. I S. 1206, 1313), work dealing with *Drosophila* and *in ovo* chick experiments does not require permission. An ethics statement therefore is not required for the work reported in this manuscript. All animal work has been conducted according to relevant national and international guidelines.

### Flies

All fly stocks were maintained and raised on standard cornmeal-yeast-agar medium. Unless otherwise noted, all fly strains used in this study are available at the Bloomington *Drosophila* stock center. The site-directed and random-inserted *UAS:TDP-43* transgenic lines were generated by germline transmission (BestGene). Strain PBac{yellow[+]-attP-3B}VK00002 (cytological region 28E7 on 2nd chromosome; strain identifier at BestGene: 9723) was used to generate the site-directed insertions of different TDP-43 variants.

### Cloning and Mutagenesis

Amino acid changes of TDP-43[WT] were introduced by site-directed mutagenesis using following primers (sequence 5′-3′):

F147L/F149L.For: TCAAAGGGGTTAGGCTTAGTTCGTTTTACGG

F147L/F149L.Rev: CCGTAAAACGAACTAAGCCTAACCCCTTTGA

G287S.For: GCTTTGGGAATCAGAGTGGATTTGGTAATA

G287S.Rev: TATTACCAAATCCACTCTGATTCCCAAAGC

A315T.For: GGGATGAACTTTGGTACGTTCAGCATTAAT

A315T.Rev: ATTAATGCTGAACGTACCAAAGTTCATCCC

G348C.For: CAGAACCAGTCATGCCCATCGGGTA

G348C.Rev: TACCCGATGGGCATGACTGGTTCTG

A382T.For: CTAATTCTGGTGCAACAATTGGTTGGGGA

A382T.Rev: TCCCCAACCAATTGTTGCACCAGAATTAG

N390D.For: GATCAGCATCCGATGCAGGGTCG

N390D.Rev: CGACCCTGCATCGGATGCTGATC

pENTR1A-TDP-43 was used as template. *in vitro* mutagenesis was performed using QuikChange® Site-Directed Mutagenesis Kit (Stratagene) according to manufacturer's manual.

#### Flies

For generation of transgenic flies, TDP-43 variants were amplified using primers introducing 5′-*Bam*HI and 3′-*Xba*I restriction sites, respectively (see below). Subsequent digest with respective enzymes allowed sub-cloning into pUASattB vector (cut with *Bgl*II and *Xba*I).

pENTR1A.For: TTCAGTCGACTGGATCCTATAGGGAG

pENTR1A.Rev: GTCTAGACTACATTCCCCAGCCAGAA

#### Human cell culture

For expression in human cells, respective TDP43 variants were subcloned in frame (*Bam*HI/*Hind*III) into pcDNA3.1(-) (Invitrogen) with an inserted 5′-Flag tag (*Not*I/*Eco*RI).

#### Chick

The *Hb9::Cre* vector was a gift from S. Pfaff. The *pCAGGS::T2TP* vector [28] was a gift from K. Kawakami. The expression vector consisted of a *pCAGGS* promoter, followed by three *SV 40 pA* signals flanked by two *loxP* sites, V5-tag TDP-43 expression cassette, *IRES* (internal ribosome entry site), *MmCherry* open reading frame and a terminating *SV40 poly-A* signal, and was inserted between the *Tol_2_*-sites as a 5′*Ssp*I/3′*Xho*I fragment. The *Tol_2_* vector [29] was a gift from A. Urasaki. The TDP-43 variants were inserted in frame between the V5-tag and the *IRES* as a 5′*Xba*I/3′*Sma*I fragment after PCR amplification using the following primers:

TDP-43 *Xba*I.For: GCTCTAGAATGTCTGAATATATTCGGGTAACC

TDP-43 *Sma*I.Rev: TCCCCCGGGCTACATTCCCCAGCCAGAAGACTT

TDP-43^CTF^ *Xba*I.For: GCTCTAGACTGCGGGAGTTCTTCT

TDP-43^NTF^ *Sma*I.Rev: CCCGGGGGACTATTAAGCATCTGTCTCATCCATTT

### Longevity

For pan-neural expression, 1^st^ chromosomal P{w[+mW.hs] = GawB}elav[C155] (*elav^C155^::Gal4*) was used to drive the different TDP-43 variants. For embryonic-onset longevity analysis, *elav^C155^::Gal4* virgins were crossed to males carrying different TDP-43 variants (*w/Y;UAS::TDP-43ICyO*). The F1 generation was raised at 25°C, male flies with driver and respective TDP-43 transgenes (*elav^C155^::Gal4/Y;UAS::TDP-43I+*) were used for analysis. Driver flies (*elav^C155^::Gal4/Y*) served as control. Statistical analysis was performed using GraphPad Prism.

In case of adult-onset expression, virgins carrying a temperature sensitive Gal80 in combination with the pan-neural driver P{w[+mW.hs] = GawB}elav[C155]; P{w[+mC] = tubP-GAL80[ts]}20 (*elav^c155^::Gal4;Gal80[ts]*) were crossed to *w/Y;UAS::TDP-43ICyO* males, respectively. The F1 generation was raised at 18°C to abolish TDP-43 expression and male flies *elav^C155^::Gal4/Y;UAS::TDP-43IGal80[ts]* were analyzed. In both paradigms, groups of 10-20 male flies (0-48 hours post hatching) per vial were shifted to 29°C, and dead flies were counted daily. Fresh food vials were provided every second day. At least 60 flies were analyzed per genotype.

### Climbing Assay

The 3^rd^ chromosomal driver w[*];;P{w[+mW.hs] = GawB}D42 (*D42::Gal4*) was used to drive motor neuron-specific expression of TDP-43 variants. Flies were raised at 25°C, male flies (*w/Y;UAS::TDP-43I+;D42::Gal4/+*) were collected (0–48 hours post hatching) and shifted to 29°C. Individual flies were tested 3 times per time point (1, 10 and 20 days after temperature shift) for negative geotaxis (ability to climb 8 cm in 10 s). At least a total number of 60 flies was used per time point and genotype. Statistical analysis was performed using GraphPad Prism.

### Western Blot

Heads of flies were dissected and homogenized in RIPA buffer (50 mM Tris pH 8.0, 150 mM NaCl, 0.1% SDS, 0.5% sodium deoxycholate, 1x protease inhibitors (Roche), 1% Nonidet P-40). Lysates were separated on 12% Gel by SDS-PAGE. Proteins were transferred to nitrocellulose membrane. Membrane was blocked with 5% (w/v) skimmed milk Tris buffered saline containing 0.1% Tween 20. For detection of proteins the following primary antibodies were used: anti-TARDBP (1:1,000, ProteinTech), anti-Syntaxin (1:2,500, Developmental Studies Hybridoma Bank (DSHB)). Secondary HRP-coupled antibodies (1:10,000, Amersham) were used for chemiluminiscence detection (Immun-Star™, WesternC™ Chemiluminescent Kit, BioRad).

### Quantitative PCR analysis

Heads of flies with pan-neural expression of different TDP-43 variants were isolated using Trizol (Invitrogen). For qRT–PCR, 1 µg total RNA was reverse transcribed using Transcriptor High Fidelity cDNA Synthesis kit (Roche) and anchored oligo-dT primer. 1/10 dilutions were used in triplicates with 0.2 µM primer and 5 µl LightCycler 480 SYBR Green I Master in a 10 µl reaction and qPCR executed in a 384-well block on a LightCycler 480 system (Roche). Amplification efficiencies of targets and references were determined and used for further calculations of normalized relative (Delta DeltaC(T)) expression levels according to [30]. One-way ANOVA with Bonferroni correction was used for statistical analysis (GraphPad Prism). Primers used for qRT-PCR amplification:

Actin-5C.For: CACACCGTGCCCATCTACGAGG

Actin-5C.Rev: CTTCTGCATACGGTCGGCGATGC

TDP-43.For: ACTGAGGATGAGCTGCGGGAGTTC

TDP-43.Rev: CAAAGAGACTGCGCAATCTGATCATCTG

TBPH.For: GGAAGGGGCCGAATAACCCGAAC

TBPH.Rev: CACACATCATTGGGTGACAGGCACC

### Cell culture

HEK293E cells (Invitrogen) were grown in Dulbecco's modified Eagle medium (DMEM), supplemented with 10% fetal bovine serum (PAA) at 37°C under humidified 5% CO_2_/air. DNA transfections were performed with FuGene6 (Roche) according to manufacturer's instruction.

### RNA crosslinking procedures

UV-crosslinking of *in vitro* transcribed biotinylated RNA to protein lysate was performed as described recently [16] with slight modifications. In brief, 1.5 µl of each sense and antisense HPLC-purified oligonucleotides (100 pmol/µl) containing a stretch of 12 TG or TC repeats 3′ of a T7 promotor sequence (TAATACGACTCACTATAGGG) were diluted in 47 µl annealing buffer (30 mM Hepes (pH 7.4), 100 mM KAc, 2 mM MgAc) and heated to 95°C for 5 min. After cooling down slowly, 12 µl of annealed oligos were *in vitro* transcribed/biotinylated using Biotin RNA Labeling Mix and T7 RNA Polymerase (both Roche) according to manufacturer's instructions. After digest of DNA, 2 µl of this reaction were incubated with 250 µl RNA binding buffer (20 mM HEPES (pH 7.5), 5 mM MgCl_2_, 50 mM KCl, 150 mM NaCl, 0.5 mM EGTA, 0.5 mM dithiothreitol, 10% glycerol) and 1,000 µg HEK293 lysate for 20 min at 30°C. HEK293 lysates were generated from HEK293 cells transfected with Flag-TDP-43 wild type or F147L/F149L for 48 h and lysed in RNA binding buffer +1% Triton X-100. After UV irradiation and brief RNase A digest, Streptavidin-beads (Sigma) were added and precipitations carried out overnight at 4°C. Eluate and input were separated by 10% SDS-PAGE, blotted onto nitrocellulose and probed overnight with antibodies against TDP-43 (1:2,000, *ProteinTech*), Flag (1:10,000, *Sigma*, clone M2) and GAPDH (1:35,000, Biodesign International) as a loading control.

### *In ovo* electroporation

Fertilized chick (*Gallus gallus*) eggs were obtained from Horstmann Geflügelzucht GmbH. Eggs were incubated at 37.5°C and 80% humidity (J. Hemel-Brutgeräte GmbH) for 72 hours (∼3 days) to obtain embryos at embryonic day 3 (E3). Microinjections were performed using a micropipette needle made from pulled glass capillary tubes. The needle was loaded with a mixture of DNA, *Hb9::Cre* (1.0 µg/µl) for motor neuron-specific expression, *pCAGGS::T2TP* (1.0 µg/µl) and *LTEV* containing the TDP-43 variant (concentration of 1.0 µg/µl). Up to 5 ml of egg white was removed using a syringe. Afterwards the egg was windowed in the middle. Egg yolk and the embryo stayed unharmed during this procedure. The DNA-mixture got injected into the neural tube (lumbar region) of the embryo. The injection site was electroporated using an ECM 830 electroporation system (BTX Harvard Apparatus, Holliston, USA). The electrodes were placed in parallel so that the developing spinal cord was situated between the electrodes. Electroporation with the electrodes placed in this way transported DNA located in the spinal cord towards the positive electrode, resulting in approximately half of the spinal cord being transfected. The electroporation settings were 5 pulses of 25 mV for 50 ms in the LV 99 ms/500 V modus. After electroporation, the window on the operated egg was sealed with clear sellotape, and the egg was returned to the incubator.

### Chick embryo and tissue processing

Chick embryos were harvested 2 and 6 days after electroporation, and placed in cold 1x PBS. Spinal cords were dissected at embryonic day 9 (E9), while E5 spinal cords were left intact in the embryo to minimize damage. Tissues were fixed by immersion in 4% PFA (in 1x PBS) (1.5–2.5 h for E5, 4–6 h for E9) and then washed in 1x PBS overnight. Afterwards the tissue was incubated in 30% sucrose (in 1x PBS) overnight. All previous steps were taken out at 4°C. Ready fixed tissue was imbedded into OCT (Jung, Nussloch). Transversal cryosections of the spinal cord were cut in a CM1900 cryostat (Leica, Bensheim) at −20°C and placed on a super frost glass slide.

### Immunohistochemistry

Chick immunofluorescent staining was performed on 30 µm slices of prefixed E5 and E9 chick embryo spinal cord. For primary antibody detection the slides were incubated in a humidified box overnight at 4°C, utilizing 1% BSA (fraction V) in 1x PBS with 0.5% Triton X-100 to enhance tissue penetration. Slides were washed with 1x PBS before the secondary staining, which was carried out with the same detergent for 30 min–1 h at RT. Slides were washed again with 1x PBS and stained with DAPI (Roth, 1∶10^6^ w/v) at RT for 10–20 min. After DAPI staining, the slides were washed with 1x PBS, mounted with 50% glycerol and cover slipped. Primary antibodies: rabbit anti-Isl1/2 (K5, 1:2,500, gift from Samuel L. Pfaff), mouse anti-Isl1 (DSHB, 1∶200), mouse anti-V5 (Invitrogen, 1∶500). Secondary antibodies: Alexa 647 rabbit anti-mouse (Invitrogen, 1∶1,000), Alexa 488 mouse anti-rabbit (Invitrogen, 1∶1,000), Alexa 647 mouse anti-rabbit (Invitrogen, 1∶1,000), Alexa 488 rabbit anti-mouse (Invitrogen, 1∶1,000). All images of mounted slices were collected using a Leica TCS/MP confocal/two-photon microscope. All images were acquired from 30 µm sections of the chick embryo spinal cord at the developmental stages of E5 and E9. Images of the E9 sections were acquired by using the 20x objective with 2x zoom. Images of the E5 sections were acquired with the 40x objective and 2x zoom. For both developmental stages (E5, E9) all images were a total of a 50–80 µm stack of 10 z-sections subsequently collapsed to a 2D rendering using ImageJ. Motor neuron survival was measured by counting Isl1/2 positive cells in the ventral horn, electroporated vs. non-electroporated side. The counting was performed using the Olympus Cell∧M imaging station.

*Drosophila* brains and eye discs were dissected in 1x PBS +0.1% Triton X-100 (PBST) and fixed in PBS +4% PFA for 20 min at RT. Primary antibody detection was performed at 4°C in PBST supplemented with 5% BSA (fraction V) over night. Tissue was washed in PBST before secondary antibodies were applied (2 h at RT). Specimen was mounted in Vectashield® mounting medium (Vector Laboratories) +/− DAPI. Primary antibodies: mouse anti-TDP-43 (1∶1,000, ProteinTech). Secondary antibodies: Alexa 488 goat-anti mouse (Invitrogen, 1∶500). Alexa Fluor 568 phalloidin (Invitrogen) was used to stain F-actin (1 unit/staining) and for confocal analysis, DNA was visualized using Sytox® Orange (Molecular Probes, 5 µM). Epifluorescent pictures were acquired at Olympus BX51 microscope (10x or 40x objectives) using Cell∧F software. Confocal sectioning was performed at Leica TCS/MP confocal/two-photon microscope using 20x objective with 2x zoom.

HEK293E cells were plated onto glass coverslips coated with PDL (Sigma) and collagen (Cohesion), fixed with 4% (w/v) paraformaldehyde and permeabilized with 1% Triton X-100 48h after transfection with Flag-tagged TDP-43 variants. Cells were incubated with primary antibodies (anti-TDP-43; 1∶2,000 and anti-Flag, 1:500; Sigma, clone M2) followed incubation with secondary antibodies anti-mouse IgG Alexa Fluor-and anti-rabbit Alexa Fluor-568 (Molecular Probes) diluted 1:2000. Nuclei were stained with Hoechst 33342 (Molecular Probes) diluted 1:5000. Coverslips were mounted onto microscope slides using fluorescent mounting medium (Dako). Confocal fluorescent images were taken with an AxioImager microscope equipped with an ApoTome Imaging System (Zeiss).

## Supporting Information

Figure S1 Localization of TDP-43 in non-neuronal cells of *Drosophila*. Salivary glands of *Drosophila* larvae were used to address subcellular localization of TDP-43 variants due to their large cell and nucleus size. Epifluorescence pictures of gland cells stained for TDP-43 (green) and f-Actin (red) using phalloidine (upper panel). Nuclear localization of TDP-43^WT^ and TDP-43^MS^ is visualized by colocalization with DAPI (blue) stained nuclei (middle panel) and illustrated by plotted fluorescence distribution (lower panel). Line indicates distance used for fluorescence measure. Note that of the assayed TDP-43 variants, only TDP-43^ΔNLS^ displayed robust cytoplasmic localization (B).
(4.10 MB TIF)

Figure S2 Subcellular localization of TDP-43:GFP variants in *Drosophila*. We were not able to efficiently detect TDP-43^CTF^ with our TDP-43 antibody directed to the N-terminal portion of the protein. To circumvent this problem, in addition to *φ-C31* site-specific recombination, we also utilized random-insertion transgenesis in *Drosophila* to generate C-terminal GFP-tagged TDP-43 variants for TDP-43^WT^ (A), TDP-43^ΔNLS^ (B) and TDP-43^CTF^ (C), (TDP-43^WT^:GFP, TDP-43^ΔNLS^:GFP and TDP-43^CTF^:GFP, respectively). This allowed us to visualize TDP-43:GFP (green) directly. First we tested the different variants in non-neuronal cells of the salivary glands of L3 larvae (left). Epifluorescence analysis revealed that GFP-tagged TDP-43^WT^ and TDP-43^ΔNLS^ localized alike the untagged variants. (A) TDP-43^WT^:GFP showed nuclear localization, evident by colocalization with DAPI stained DNA (blue). (B) TDP-43^ΔNLS^:GFP was found to evenly distributed in the cytoplasm and almost not detected in the nucleus. Thus, the C-terminal GFP-tag did not alter localization, as it was also observed in HEK cells (not shown). (C) TDP-43^CTF^:GFP showed a strong cytoplasmic as well as a nuclear localization, similar to the situation observed in chick (compare to Fig S3A). In *Drosophila* larvae, photoreceptors and their nuclei are located in the eye imaginal disc, from where photoreceptors project via the optic nerve into the optic lobes of the brain. We used the visual system to analyze the distribution of the TDP-43:GFP variants in these neuronal cells (right). Expression of all three constructs in photoreceptors (*GMR::Gal4*) resulted in a robust accumulation of GFP signal in targeted cells of the eye disc (not shown). As expected, larvae expressing TDP-43^WT^:GFP did not display any GFP signal in photoreceptor projections (A), as localization was restricted to the nuclei of the eye disc (see also Fig. 3A). In contrast, we could detect robust staining in photoreceptor projections in TDP-43^ΔNLS^:GFP and TDP-43^CTF^:GFP (B, C). This indicates that cytoplasmic ΔNLS and CTF variants of TDP-43:GFP were distributed throughout neurons and were not restricted to the soma. Scale bar indicates 100 µm.
(2.36 MB TIF)

Figure S3 Subcellular localization of additional TDP-43 variants in *Gallus*. Subcellular localization of TDP-43 variants (red) in E9 chick motor neurons (large DAPI+ nuclei: white). In analogy to cytosolic and nuclear localization observed in flies, TDP-43^CTF^ (A) in chick localized alike and displayed frequent cytosolic foci (arrowheads). To test if the lack of RNA-binding also abolishes toxicity of cytoplasmic TDP-43, we combined RNA-binding deficient FFLL with the mutated NLS and generated TDP-43^ΔNLS,FFLL^. As observed for TDP-43^ΔNLS^ (compare Fig. 3B), TDP- 43^ΔNLS,FFLL^ localized predominantly to the cytoplasm (B). Thus, interference with RNA-binding of TDP-43 did not alter localization. Interestingly, TDP-43^ΔNLS,FFLL^ was found to induce significantly lower Caspase-3 activation as compared to TDP-43^ΔNLS^ (see Fig. 5Cii). This indicated that RNA-binding of TDP-43 is required to mediate neurotoxicity, independent of nuclear or cytoplasmic localization. TDP-43^CTF^ was found to induce comparatively mild, if any toxicity in our in vivo systems (see Fig. 4 and Fig. 5B, C). As TDP-43^CTF^ lacks the first RRM, found to be crucial to mediate TDP-43 induced toxicity, we reasoned that the N-terminal fragment TDP-43^NTF^ (amino acids 1-263, containing RRM1/2) might display marked effects after expression in chick motor neurons. Although we achieved high expression levels and nuclear localization of TDP-43^NTF^ in motor neurons (C), we could not detect elevated motor neuron loss (see Fig. 5B) or enhanced Caspase activation compared to vector control (see Fig. 5C). Scale bar indicates 50 µm.
(4.86 MB TIF)

Figure S4 Reduction in longevity is dose-dependent and inducible in an adult-onset expression system. (A) Two TDP-43^WT^:GFP lines displaying different expression levels in Western blot analysis were assayed for longevity after pan- neural expression. The line with stronger expression, TDP-43^WT^:GFP#16 displayed an earlier lethality as compared to the weaker expressing line TDP-43^WT^:GFP#10. This indicates that toxicity induced by pan-neural expression of TDP-43^WT^ is dose-dependent. According to the GFP-Tag, we were able to compare protein abundance of different TDP-43^CTF^:GFP transgenes. Compared to TDP-43^WT^:GFP#10, TDP-43^CTF^:GFP#14 showed higher levels of protein expression. In spite of these high protein levels, longevity was not as strongly reduced as observed for TDP-43^WT^:GFP expressing flies. (B) Verification of pan-neural adult-onset expression in Western blot analysis. Flies were raised at restrictive condition (rc, 18°C), and TDP-43 expression was induced 2 days post eclosion by a temperature shift (29°C). Head lysates taken at indicated time points were analyzed for TDP-43 expression. No TDP-43^WT^:GFP expression could be detected under rc, but was induced at 29°C. Increasing TDP-43 protein levels were detected over time. (C) Flies with adult-onset pan-neural expression of indicated TDP-43 variants were assayed for longevity. Note that line TDP-43^WT^:GFP#14, lethal in embryonic-onset expression paradigm, survived to adulthood in the adult-onset expression, but displayed a very short longevity after induction of expression. For untagged and site-directed TDP-43 transgenes, the order of toxicity is comparable to the embryonic-onset paradigm (see Fig. 4).
(0.55 MB TIF)

Video S1 Abnormal locomotion and wing posture in aged TDP-43^WT^ expressing flies. Example of a fly with pan-neural expression of TDP-43^WT^. Note the lateral “hanging wing” phenotype. Dependent on expression strength and time, TDP-43^WT^ flies lost their ability to fly, had reduced escape behavior (even if pushed with a brush) and were not able to reach an upright position after being placed dorsal side down in a reasonable time.
(1.35 MB MOV)
