# Supplementary figures and images for: TDP-43-Mediated Neuron Loss In Vivo Requires RNA-Binding Activity
Source: PLoS One. 2010 Aug 18;5(8):e12247. doi: 10.1371/journal.pone.0012247 (PMC2923622; doi:10.1371/journal.pone.0012247)

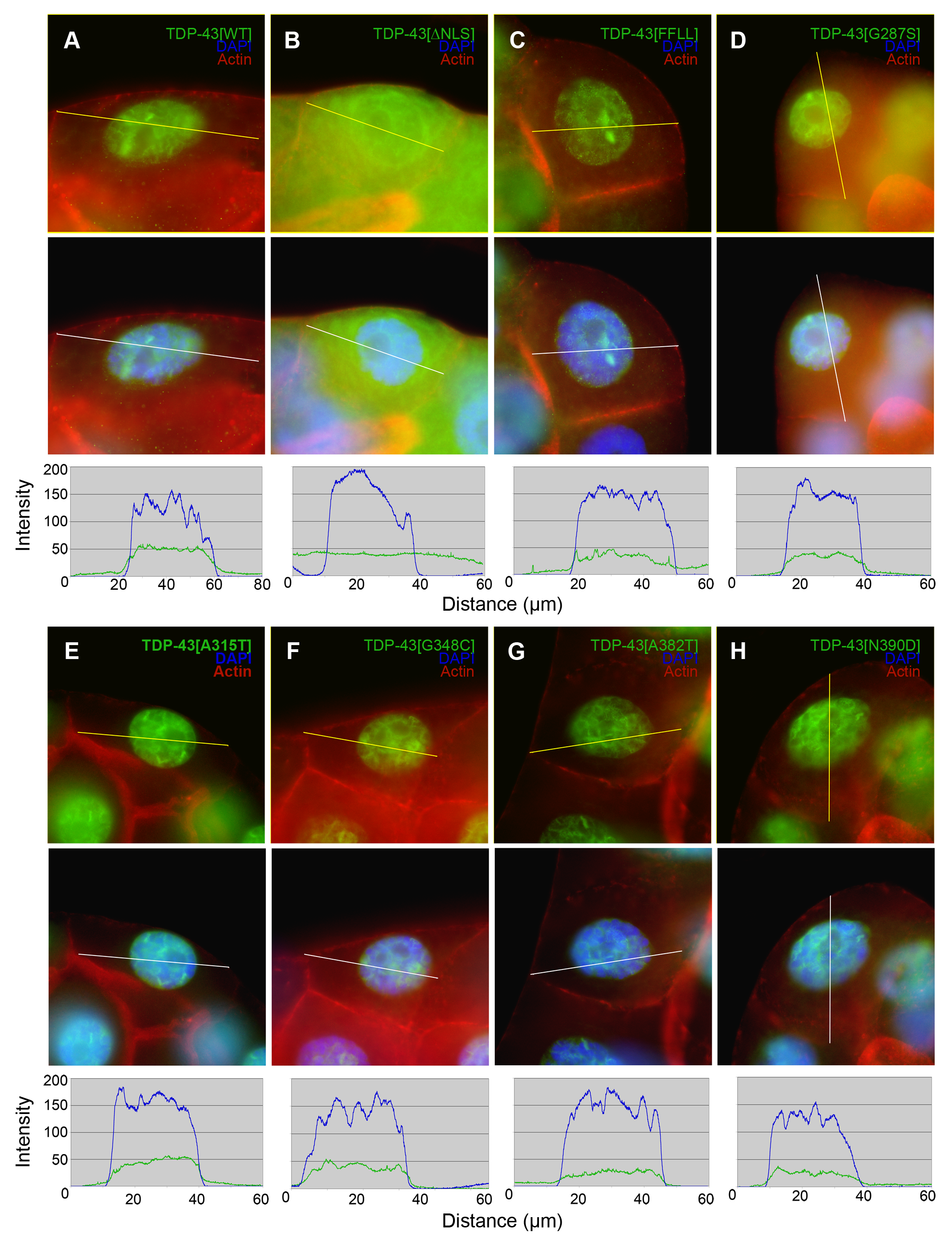

Supplement: Figure S1 — Localization of TDP-43 in non-neuronal cells of Drosophila. Salivary glands of Drosophila larvae were used to address subcellular localization of TDP-43 variants due to their large cell and nucleus size. Epifluorescence pictures of gland cells stained for TDP-43 (green) and f-Actin (red) using phalloidine (upper panel). Nuclear localization of TDP-43WT and TDP-43MS is visualized by colocalization with DAPI (blue) stained nuclei (middle panel) and illustrated by plotted fluorescence distribution (lower panel). Line indicates distance used for fluorescence measure. Note that of the assayed TDP-43 variants, only TDP-43ΔNLS displayed robust cytoplasmic localization (B). (4.10 MB TIF) [file pone.0012247.s001.tif]

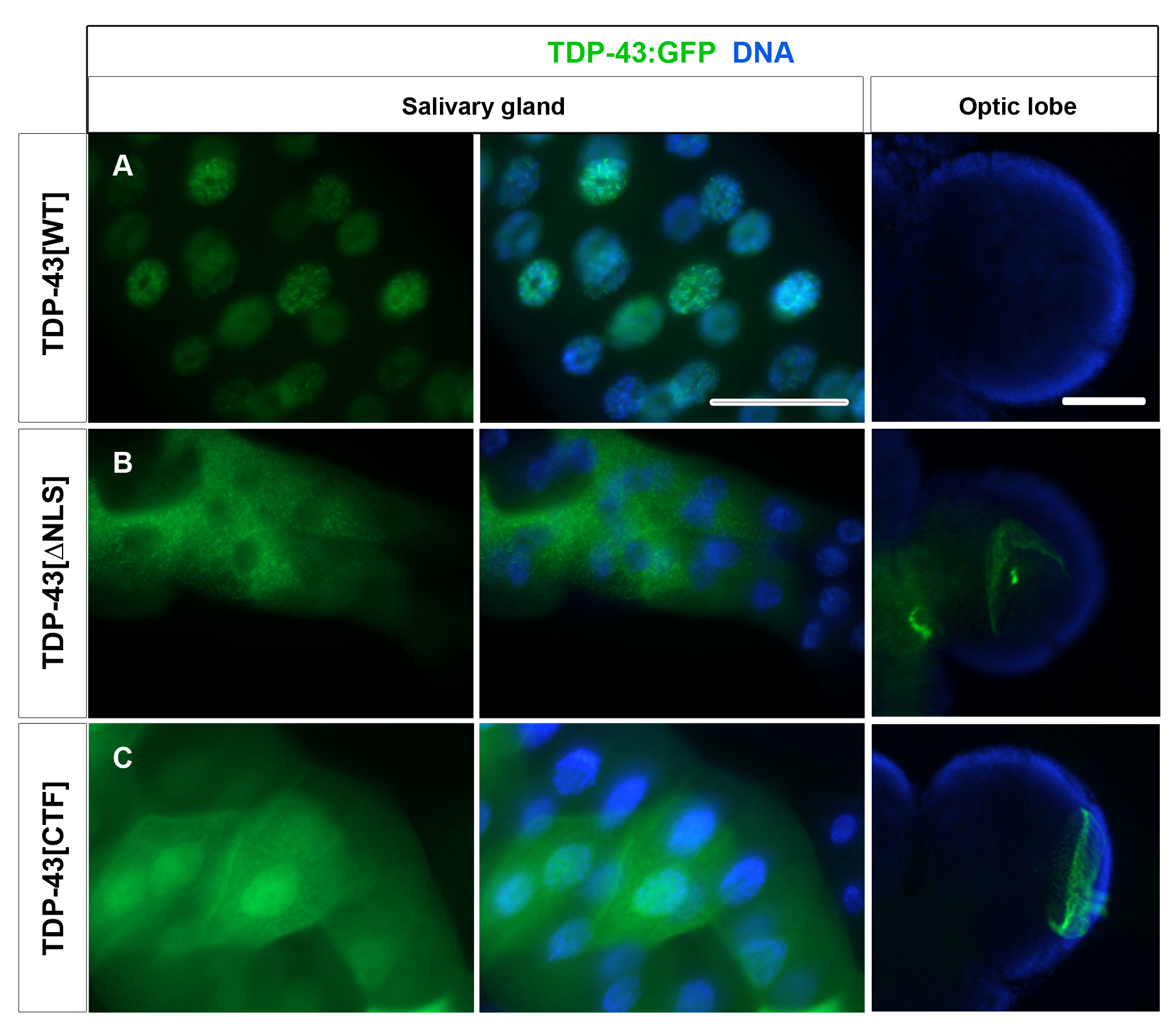

Supplement: Figure S2 — Subcellular localization of TDP-43:GFP variants in Drosophila. We were not able to efficiently detect TDP-43CTF with our TDP-43 antibody directed to the N-terminal portion of the protein. To circumvent this problem, in addition to φ-C31 site-specific recombination, we also utilized random-insertion transgenesis in Drosophila to generate C-terminal GFP-tagged TDP-43 variants for TDP-43WT (A), TDP-43ΔNLS (B) and TDP-43CTF (C), (TDP-43WT:GFP, TDP-43ΔNLS:GFP and TDP-43CTF:GFP, respectively). This allowed us to visualize TDP-43:GFP (green) directly. First we tested the different variants in non-neuronal cells of the salivary glands of L3 larvae (left). Epifluorescence analysis revealed that GFP-tagged TDP-43WT and TDP-43ΔNLS localized alike the untagged variants. (A) TDP-43WT:GFP showed nuclear localization, evident by colocalization with DAPI stained DNA (blue). (B) TDP-43ΔNLS:GFP was found to evenly distributed in the cytoplasm and almost not detected in the nucleus. Thus, the C-terminal GFP-tag did not alter localization, as it was also observed in HEK cells (not shown). (C) TDP-43CTF:GFP showed a strong cytoplasmic as well as a nuclear localization, similar to the situation observed in chick (compare to Fig S3A). In Drosophila larvae, photoreceptors and their nuclei are located in the eye imaginal disc, from where photoreceptors project via the optic nerve into the optic lobes of the brain. We used the visual system to analyze the distribution of the TDP-43:GFP variants in these neuronal cells (right). Expression of all three constructs in photoreceptors (GMR::Gal4) resulted in a robust accumulation of GFP signal in targeted cells of the eye disc (not shown). As expected, larvae expressing TDP-43WT:GFP did not display any GFP signal in photoreceptor projections (A), as localization was restricted to the nuclei of the eye disc (see also Fig. 3A). In contrast, we could detect robust staining in photoreceptor projections in TDP-43ΔNLS:GFP and TDP-43CTF:GFP (B, C). This [file pone.0012247.s002.tif]

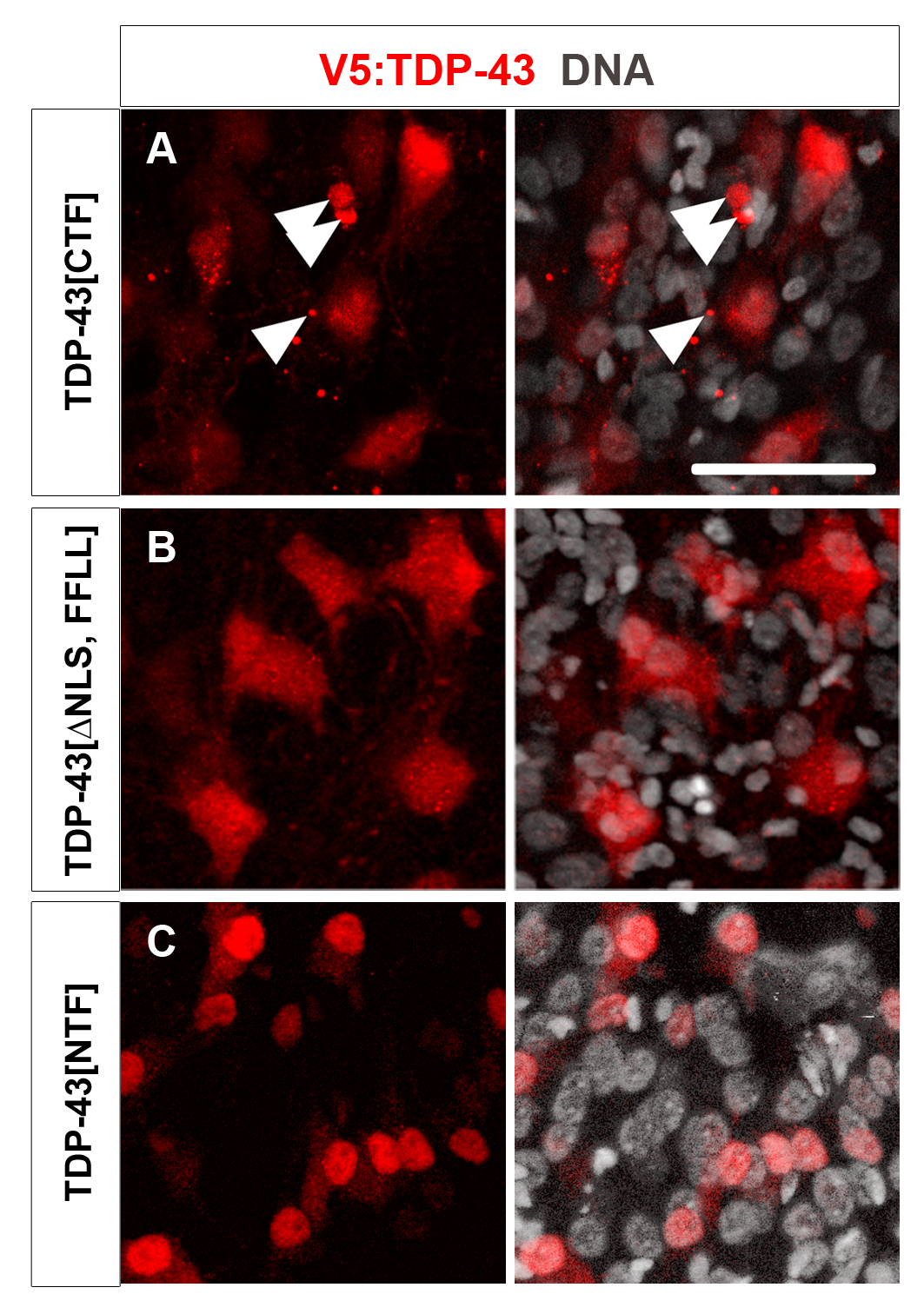

Supplement: Figure S3 — Subcellular localization of additional TDP-43 variants in Gallus. Subcellular localization of TDP-43 variants (red) in E9 chick motor neurons (large DAPI+ nuclei: white). In analogy to cytosolic and nuclear localization observed in flies, TDP-43CTF (A) in chick localized alike and displayed frequent cytosolic foci (arrowheads). To test if the lack of RNA-binding also abolishes toxicity of cytoplasmic TDP-43, we combined RNA-binding deficient FFLL with the mutated NLS and generated TDP-43ΔNLS,FFLL. As observed for TDP-43ΔNLS (compare Fig. 3B), TDP- 43ΔNLS,FFLL localized predominantly to the cytoplasm (B). Thus, interference with RNA-binding of TDP-43 did not alter localization. Interestingly, TDP-43ΔNLS,FFLL was found to induce significantly lower Caspase-3 activation as compared to TDP-43ΔNLS (see Fig. 5Cii). This indicated that RNA-binding of TDP-43 is required to mediate neurotoxicity, independent of nuclear or cytoplasmic localization. TDP-43CTF was found to induce comparatively mild, if any toxicity in our in vivo systems (see Fig. 4 and Fig. 5B, C). As TDP-43CTF lacks the first RRM, found to be crucial to mediate TDP-43 induced toxicity, we reasoned that the N-terminal fragment TDP-43NTF (amino acids 1-263, containing RRM1/2) might display marked effects after expression in chick motor neurons. Although we achieved high expression levels and nuclear localization of TDP-43NTF in motor neurons (C), we could not detect elevated motor neuron loss (see Fig. 5B) or enhanced Caspase activation compared to vector control (see Fig. 5C). Scale bar indicates 50 µm. (4.86 MB TIF) [file pone.0012247.s003.tif]

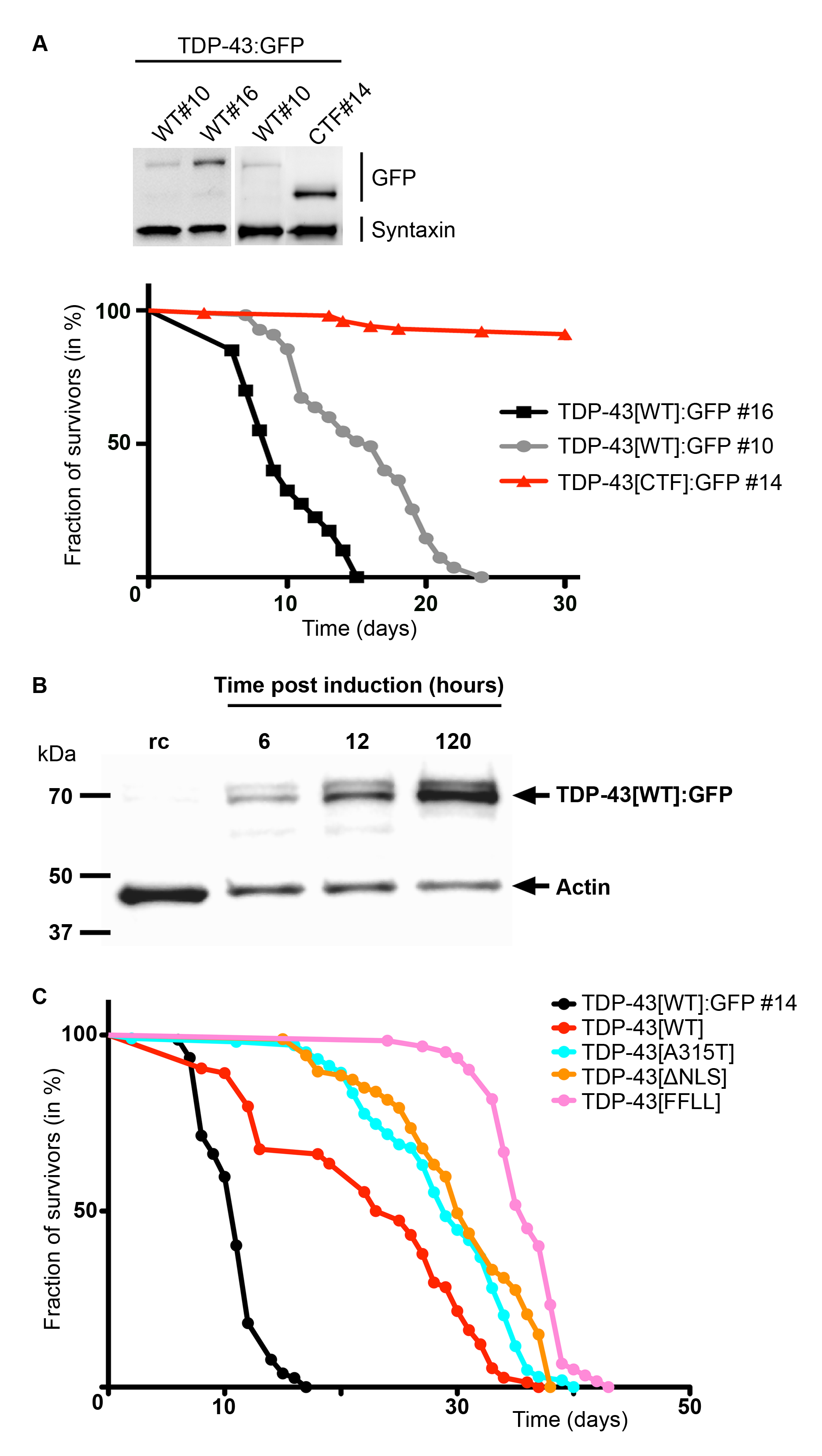

Supplement: Figure S4 — Reduction in longevity is dose-dependent and inducible in an adult-onset expression system. (A) Two TDP-43WT:GFP lines displaying different expression levels in Western blot analysis were assayed for longevity after pan- neural expression. The line with stronger expression, TDP-43WT:GFP#16 displayed an earlier lethality as compared to the weaker expressing line TDP-43WT:GFP#10. This indicates that toxicity induced by pan-neural expression of TDP-43WT is dose-dependent. According to the GFP-Tag, we were able to compare protein abundance of different TDP-43CTF:GFP transgenes. Compared to TDP-43WT:GFP#10, TDP-43CTF:GFP#14 showed higher levels of protein expression. In spite of these high protein levels, longevity was not as strongly reduced as observed for TDP-43WT:GFP expressing flies. (B) Verification of pan-neural adult-onset expression in Western blot analysis. Flies were raised at restrictive condition (rc, 18°C), and TDP-43 expression was induced 2 days post eclosion by a temperature shift (29°C). Head lysates taken at indicated time points were analyzed for TDP-43 expression. No TDP-43WT:GFP expression could be detected under rc, but was induced at 29°C. Increasing TDP-43 protein levels were detected over time. (C) Flies with adult-onset pan-neural expression of indicated TDP-43 variants were assayed for longevity. Note that line TDP-43WT:GFP#14, lethal in embryonic-onset expression paradigm, survived to adulthood in the adult-onset expression, but displayed a very short longevity after induction of expression. For untagged and site-directed TDP-43 transgenes, the order of toxicity is comparable to the embryonic-onset paradigm (see Fig. 4). (0.55 MB TIF) [file pone.0012247.s004.tif]
